# Use of wearable biometric monitoring devices to measure outcomes in randomized clinical trials: a methodological systematic review

**DOI:** 10.1186/s12916-020-01773-w

**Published:** 2020-11-06

**Authors:** Carolina Graña Possamai, Philippe Ravaud, Lina Ghosn, Viet-Thi Tran

**Affiliations:** 1 METHODS Team, Center for Research in Epidemiology and Statistics (CRESS), Université de Paris/INSERM (UMR 1153), 1 Place du Parvis Notre Dame, 75004 Paris, France; 2 Centre d’Epidémiologie Clinique, Hôpital Hôtel-Dieu (AP-HP), 1 Place du Parvis Notre Dame, 75004 Paris, France; 3 Department of Epidemiology, Columbia University Mailman School of Public Health, 22 W 168th St, New York, NY USA

**Keywords:** Clinical trials, Outcomes, Wearable devices

## Abstract

**Background:**

Wearable biometric monitoring devices (BMDs) have the potential to transform the conduct of randomized controlled trials (RCTs) by shifting the collection of outcome data from single measurements at predefined time points to dense continuous measurements.

**Methods:**

Methodological systematic review to understand how recent RCTs used BMDs to measure outcomes and to describe the reporting of these RCTs. Electronic search was performed in the Cochrane Central Register of Controlled Trials, PubMed, and EMBASE and completed a page-by-page hand search in five leading medical journals between January 1, 2018, and December 31, 2018. Three reviewers independently extracted all primary and secondary outcomes collected using BMDs, and assessed (1) the definitions used to summarize BMD outcome data; (2) whether the validity, reliability, and responsiveness of sensors was reported; (3) the discrepancy with outcomes prespecified in public clinical trial registries; and (4) the methods used to manage missing and incomplete BMD outcome data.

**Results:**

Of the 4562 records screened, 75 RCTs were eligible. Among them, 24% tested a pharmacological intervention and 57% used an inertial measurement sensor to measure physical activity. Included trials involved 464 outcomes (average of 6 [SD = 8] outcomes per trial). In total, 35 trials used a BMD to measure a primary outcome. Several issues affected the value and transparency of trials using BMDs to measure outcomes. First, the definition of outcomes used in the trials was highly heterogeneous (e.g., 21 diabetes trials had 266 outcomes and 153 had different unique definitions to measure diabetes control), which limited the combination and comparison of results. Second, information on the validity, reliability, and responsiveness of sensors used was lacking in 74% of trials. Third, half (53%) of the outcomes measured with BMDs had not been prespecified, with a high risk of outcome reporting bias. Finally, reporting on the management of incomplete outcome data (e.g., due to suboptimal compliance with the BMD) was absent in 68% of RCTs.

**Conclusions:**

Use of BMDs to measure outcomes is becoming the norm rather than the exception in many fields. Yet, trialists need to account for several methodological issues when specifying and conducting RCTs using these novel tools.

## Background

Biometric monitoring devices (BMDs) are sensors embedded in smartphones, wearable technologies (e.g., wrist bands, skin patches), or everyday objects (e.g., smart cap bottles) that offer the opportunity to collect biological, physiological, or behavioral patient data, continuously, remotely, and unobtrusively [1]. BMDs represent a revolution in clinical research and trials by allowing for the transition from single measurements at predefined time points (e.g., single measures of a quality of life questionnaire or a biomarker, at a given time point after randomization) to dense minute-by-minute measurements during the whole course of the trial. This new type of outcome data offers researchers the ability to precisely examine the effects of experimental treatments and interventions over time [2, 3]. In addition, BMDs permit the measurement of health, disease progression, and treatment effects in real-life situations, from patients’ homes and from widely dispersed participants living in distant locations and/or for whom mobility is limited [2].

As a result, clinical trials are starting to use BMDs to assess outcomes in addition or as a replacement to traditional measures [4, 5]. In recent diabetes trials, outcomes based on continuous glucose monitoring devices are replacing the traditional measurement of HbA1c [6]. In oncology and neurology, actigraphy and accelerometers are increasingly being used as alternatives to patient-reported questionnaires assessing physical activity or sleep quality [7–9]. The US Food and Drug Administration recently issued its Real World Evidence Program, which includes a reflection on the use of BMDs to measure novel outcomes for assessing health, disease progression, and treatment effect [10–12].

Yet, the use of BMDs to measure outcomes in clinical research is an emerging field and the challenges associated with their use remain largely unknown. As of today, the literature has focused on concerns about data validity, accuracy, provenance, and regulatory issues [4, 7, 13]. To our knowledge, only one study systematically examined the outcomes measured with activity monitors in oncology trials and found a lack of standardization in the types of BMDs used and how their data were collected, analyzed, and interpreted [14]. Other problems such as how to manage incomplete BMD outcome data (e.g., due to poor compliance) have been underlined in some trials [15]. However, conventional data imputation strategies are not adapted to manage the temporal variations in multivariate time series associated with these types of data [3, 16].

Our goal was to understand how recent RCTs used BMDs to measure outcomes and to describe the reporting of these RCTs. Especially, we aimed to answer four questions: (1) Which outcomes are measured with BMDs in trials? (2) What is the validity, reliability, and responsiveness of measurement instruments used? (3) Were outcomes measured with BMDs prespecified? and (4) How were missing and incomplete BMD outcome data managed in these RCTs?

## Methods

### Eligibility criteria

We included reports of RCTs involving adults (aged ≥ 18 years old), from any country or setting, that were published in English and used BMDs to measure outcomes. We defined BMDs as any sensor, wearable or not, that provided information on patients’ health, physiology, or behavior without any involvement of patients in measurement. For example, we did not include reports of trials that used outcomes collected by using smartphone apps that prompted patients with questions or patient self-reported data obtained from wearable devices. We excluded reports of trials with outcomes measured by BMDs during surgery or intensive care and trials of children or healthy participants. We also excluded systematic reviews or meta-analyses, diagnostic studies, methodological publications, editorial-style reviews, abstracts and posters, and secondary analyses of trials.

### Search and selection of trials

We searched PubMed, CENTRAL, and EMBASE for reports of all RCTs of any phase that used a BMD to measure at least one outcome, with publication dates between January 1, 2018, and December 31, 2018. A first set of search equations were developed for each database and were based on Medical Subject Headings (MeSH) terms and specific free-text words pertaining to wearable devices and continuous measurements (Supplementary Appendix 1a). This search was conducted on February 4, 2019. During peer-review of this article, we complemented our initial search with several additional MeSH terms (Supplementary Appendix 1b). The second search was conducted on June 26, 2020. To avoid missing trials because (1) of no validated search strategy to retrieve trials using BMDs as outcomes and (2) the names used to describe BMDs are ever-changing (sensors, wearables, trackers, continuous monitoring devices, etc.), we supplemented our search by a page-by-page hand search for eligible trials in the *New England Journal of Medicine*, the *Lancet*, the *Journal of the American Medical Association*, the *British Medical Journal*, and *Annals of Internal Medicine*. For duplicate publications (i.e., publication of the same study by the same authors without modification of methods or results), we kept the most recent publication.

One reviewer (CG) conducted the electronic database searches and another (LG) conducted the page-by-page hand search. Then, two reviewers (CG, LG) screened articles by using Rayyan [17]. First, they examined titles and abstracts (when available) and selected full-text articles according to the specified eligibility criteria. If an abstract was not provided by the database it originated from, and the title appeared to be potentially relevant, we reviewed its full text. A third investigator (VTT) verified 20% of included studies and 10% of excluded studies, with agreement of 97.5%. At each stage, we recorded the records retrieved and excluded.

### Data review and extraction

#### Characteristics of included trials

For each trial, 2 reviewers (CG, VTT) independently extracted data from studies by using a standardized form. They recorded the trial characteristics (journal name; date of publication; medical area; number of randomized patients; funding source, types of interventions [i.e., pharmacological or non-pharmacological] and the design of the trial). Then, they extracted all outcomes measured with BMDs and classified them as primary or secondary outcomes. We considered primary outcomes as those that were explicitly reported as such in the published article or in the entry in a public clinical trial registry or, if none was explicitly reported, the outcome(s) that was stated in the sample size estimation. All other outcomes were considered secondary outcomes.

#### The outcomes measured using BMDs in trials

Two reviewers (CG/LG, VTT) independently described each outcome by using a framework inspired by Zarin et al. [18]. This framework was chosen because it represents the expected standard required to detect a change between prespecified and published outcomes. Outcomes were defined by their (1) concept of interest (e.g., diabetes control, mobility, pain, sleep quality, etc.) [10], (2) domain (e.g., glycemic variability), (3) specific measurement (e.g., number of hypoglycemic episodes), (4) time window during which data were collected (e.g., during the night between 8.00 PM and 6.00 AM), and (5) method to aggregate data (e.g., mean number of hypoglycemic episodes per patient). Results were synthesized by presenting all outcome definitions used to measure the same domain, by concept of interest.

#### The validity, reliability, and responsiveness of measurement instruments used

For each outcome, two reviewers extracted the precise sensor used and its type (inertial measurement units [e.g., accelerometers]; optical sensors [e.g., photoplethysmography], electrochemical sensors [e.g., continuous glucose monitoring], pressure sensors [e.g., blood pressure monitoring], temperature sensors and electrodes [e.g., electrocardiographic monitoring]). Then, they evaluated the information reported in the published articles regarding the validity, reliability, and responsiveness of sensors. Information was classified as (1) reported and documented (e.g., with references or information in supplementary materials), (2) described as “valid” or “reliable” without any further detail, or (3) missing.

#### Prespecification of outcomes measured with BMDs

Two reviewers (LG, VTT) independently compared the outcomes reported in the methods section of published articles with those registered in the latest entry in a public trial registry (e.g., ClinicalTrials.gov), searched in January 2020. Trial registration numbers were extracted from articles. When no trial registration information was found, the trial was considered unregistered. Outcome definitions could be considered “similar” (the two outcome definitions were strictly similar), “modified” (the two outcome definitions had similar domains but different measurement or thresholds), or “added” (the outcome was present in the published article but not in the public trial registry entry).

#### Management of missing and incomplete BMD outcome data in RCTs

In each included trial, two reviewers (CG/LG, VTT) independently extracted information on the management of missing and incomplete BMD outcome data. They distinguished 2 situations:

- The data were “missing.” There was no estimate of the treatment effect for the given patient (e.g., the device malfunctioned or the patient did not wear the BMD at all). To assess the management of missing data, the reviewers searched the methods section of articles for classical methods used to handle missing data (e.g., last observation carried forward, complete-case analysis, multiple imputation methods) [19]. If no specific method was found, the reviewers evaluated whether the analysis was performed on only complete cases by examining the number of patients analyzed for each outcome. If analysis was performed as intention-to-treat and no method to manage missing data was reported, they considered the method to be “unclear.”
- The data were “incomplete”. There was some information on the treatment effect for the given patient but it may be limited (e.g., participants had suboptimal compliance with the BMD during the trial, which resulted in intervals during which no data were captured). Because of no consensus on methods to account for incomplete outcome data [20], the reviewers assessed (1) how incomplete BMD outcome data were defined by authors, (2) methods to account for incomplete data, and (3) the amount of incomplete BMD outcome data present in trials.

### Analysis

Data are presented as number (percentage) for qualitative data and median [interquartile range (IQR)] for continuous data. All analyses involved using R v3.6.1 (http://www.R-project.org), the R Foundation for Statistical Computing, Vienna, Austria.

## Results

**Fig. 1:**
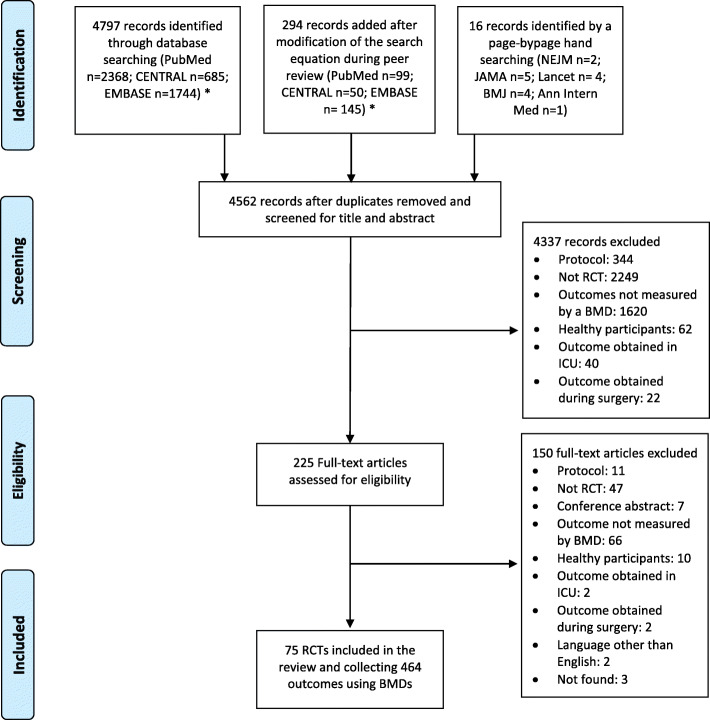
Study flow chart. * initial search was conducted on February 4, 2019. During peer review, we modified the search equation by adding several new terms. During this second search, conducted on June 26, 2020, we screened and included all eligible trials not included in the initial search

The electronic search identified 5091 records. We identified 225 records on the basis of the title and abstract and 15 by our page-by-page hand search. Finally, we analyzed 75 reports of RCTs using BMDs to measure outcomes (Fig. 1, Appendices 1 and 2).

### Characteristics of included trials

The 75 RCTs included in the review involved 10,822 patients (mean sample size 144 [SD = 322]) with various conditions such as diabetes (*n* = 25), cardiac or vascular diseases (including stroke) (*n* = 21), or cancer (*n* = 4) (Table 1). One quarter of trials (*n* = 18, 24%) tested a pharmacological intervention. Trials had mainly been conducted in Europe (*n* = 36, 48%), North America (*n* = 20, 27%), and Asia (*n* = 15, 20%).

**Table 1:** Characteristics of the included randomized controlled trials that used biometric monitoring devices (BMDs) for measuring outcomes (*n* = 75)

Characteristic	Trials, no. (%)
Study design	
Parallel	56 (75)
Cross-over	15 (20)
Cluster	3 (4)
Factorial	1 (1)
Intervention assessed	
Non-pharmacological	57 (76)
Pharmacological	18 (24)
Number of patients randomized, mean (SD)	144 (322)
Region of the primary author	
Europe	36 (48)
North America	20 (27)
Asia	15 (20)
South America	2 (3)
Africa	1 (1)
Oceania	1 (1)
Funding	
Public	64 (85)
Private or mixed funding	7 (9)
Not reported	4 (5)
Medical condition investigated in the study	
Diabetes	25 (33)
Cardiac and vascular diseases (incl. hypertension)	21 (28)
Chronic obstructive pulmonary disease	6 (8)
Cancer	4 (5)
Insomnia	4 (5)
Renal disease	2 (3)
HIV/AIDS	2 (3)
Obstructive sleep apnea	2 (3)
Osteoarthritis/osteoporosis	2 (3)
Psychiatric disorders	2 (3)
Multiple sclerosis	1 (1)
Psoriasis	1 (1)
Rheumatoid arthritis	1 (1)
Obesity	1 (1)
Spinal cord injury	1 (1)
Outcomes measured with BMDs per trial, mean (SD), no.	6 (8)
Type of sensor used*	
Inertial measurement unit sensors	43 (57)
Electrochemical sensors (including continuous glucose monitoring)	21 (28)
Pressure sensors (including smart cap bottles)	6 (8)
Electrodes	4 (5)
Temperature sensors	2 (3)
Optical sensor	2 (3)
Management of missing BMD outcome data	
Unclear	26 (35)
Exclusion of patients with missing outcome data	25 (33)
Multiple imputation	8 (11)
Use of models robust for missing data	7 (9)
Last observation carried forward	2 (3)
Value inferred by investigator	2 (3)
Missing values considered as failures	2 (3)
Other	3 (4)
Reporting on management of incomplete BMD outcome data	24 (32)

*Exceeds 100% because some trials used multiple sensors

The included trials measured a total of 464 outcomes using BMDs (64 primary outcomes and 400 secondary outcomes), with a mean of 6 (SD = 8) outcomes measured with BMDs per trial. A total of 35 (47%) trials used a BMD to measure the primary outcome (difference with the total number of primary outcomes is due to a single study that had 19 primary outcomes [21]) (Tables 2 and 3). Among the 464 outcomes, 112 (24%) were presented in the results section of articles without having been described/defined in the methods section.

**Table 2:** Characteristics of outcomes measured using BMDs according to outcome type (*n* = 464). Primary outcomes were those that were explicitly reported as such in the published article or in the entry in a public clinical trial registry or, if none was explicitly reported, the outcome(s) stated in the sample size estimation. All other outcomes were considered secondary outcomes

	Primary outcomes (***n*** = 64)	Secondary outcomes (***n*** = 400)
Type of sensor used		
Inertial measurement unit sensors	25 (39)	108 (27)
Electrochemical sensors	29 (45)	237 (59)
Pressure sensors (including smart cap bottles)	3 (5)	35 (9)
Optical sensor	0 (0)	10 (2)
Electrodes	5 (8)	9 (2)
Temperature sensors	2 (3)	1 (0.2)
Concept of interest assessed		
Diabetes control	29 (45)	237 (59)
Assessment of diabetic foot complications	0 (0)	2 (0.5)
Physical activity	19 (30)	68 (17)
Blood pressure control	3 (5)	29 (7)
Adherence to treatment	6 (9)	16 (4)
Heart rate variability	5 (8)	9 (2)
Pulmonary capacity	0 ()	2 (0.5)
Sleep disturbance	2 (3)	37 (9)
Prespecification of the outcome*	42 (66)	139 (34)

*Prespecification was assessed by looking, for each included trial, for the corresponding entry in a public clinical trial registry (e.g., clinicaltrials.gov) by looking for the trial registration number reported in the articles on January 2020 (and June 2020 for articles added during peer-review)

**Table 3:** Characteristics of trials that used an outcome measured with BMDs as a primary outcome (*n* = 75). Primary outcomes were those that were explicitly reported as such in the published article or in the entry in a public clinical trial registry or, if none was explicitly reported, the outcome(s) stated in the sample size estimation. All other outcomes were considered secondary outcomes

	Trials with at least one primary outcome measured with BMDs (***n*** = 35)	Trials with no primary outcome measured with BMDs (***n*** = 40)
Number (%) of trials reporting the validity, reliability and responsiveness of the sensor	8 (23)	12 (30)
Number (%) of trials reporting adequate methods to manage missing outcome data	19 (54)	30 (75)
Number (%) of trials reporting information on incomplete BMD outcome data	16 (46)	8 (20)

### The outcomes measured using BMDs in trials

**Fig. 2:**
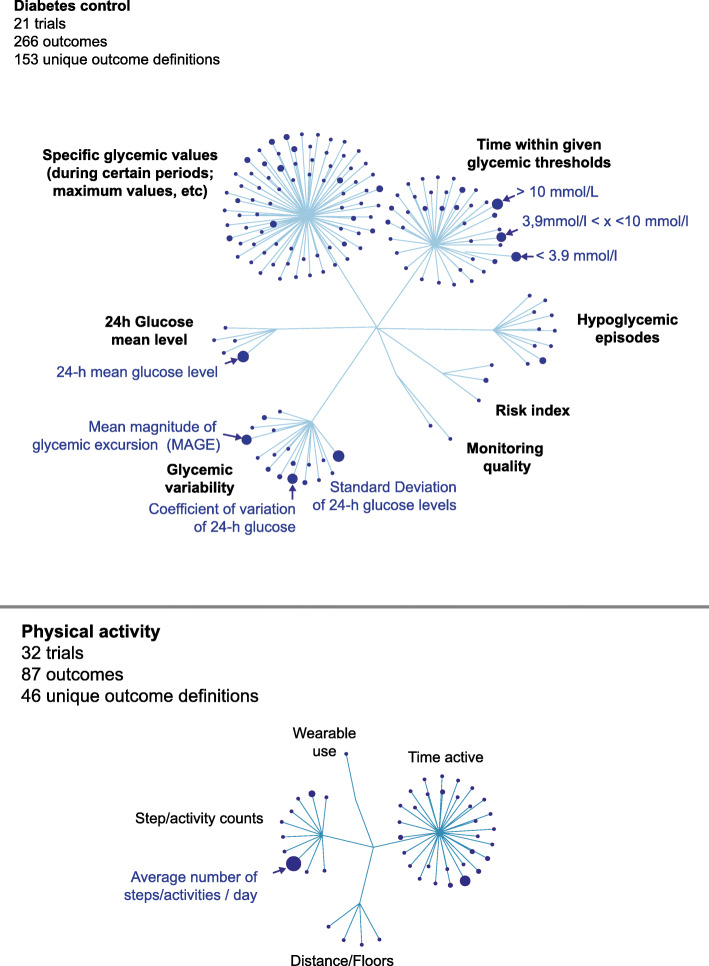
Outcome definitions in randomized controlled trials that used biometric monitoring devices (BMDs), the example of diabetes control (*n* = 21 trials with 266 outcomes and 153 unique outcome definitions), and physical activity (*n* = 32 trials with 87 outcomes and 46 unique outcome definitions). Each node represents a given outcome definition characterized by its domain, measurement method, metric, aggregation method, and time frame. The size of nodes represents the number of times each outcome definition was used in the included trials. Outcome definitions are clustered by outcome domains

We found substantial heterogeneity in the definition of outcomes used to measure the same concept of interest, with 153 different unique outcome definitions for glycemic control (of 266 outcomes), 2 for the assessment of diabetic foot complications (of 2 outcomes), 46 for physical activity (of 87 outcomes), 13 for blood pressure control (of 32 outcomes), 20 for sleep quality (of 39 outcomes), 13 for adherence to treatment (of 22 outcomes), 2 for pulmonary capacity (of 2 outcomes), and 14 for heart rate variability (of 14 outcomes) (Appendix 3). Heterogeneity in outcome definitions was due to varying combinations of domains, time frames, or algorithm used to process the raw data (Fig. 2 and Appendix 4). For example, in diabetes trials, thresholds used for glucose target range could be 3.9 to 10 mmol/L or 5.6 to 10 mmol/L depending on the study. Details of the 252 unique definitions of outcomes measured with BMDs are in Appendix 5.

### The validity, reliability, and responsiveness of measurement instruments used

Sensors most frequently used in the included trials were inertial measurement unit sensors (57%, *n* = 43) and continuous glucose-monitoring systems (28%, *n* = 21) (Table 1). Most trials (*n* = 69, 92%) reported the precise name and version of the sensor used, but only 20 (26%) provided information on the validity, reliability, and responsiveness of measurements.

### Prespecification of outcomes measured using BMDs

For the 75 included trials, we found an entry in a public trial registry for 52 (69%, 392 outcomes). For one trial, the registration number provided in the published article linked to a different study. Therefore, we compared 385 outcome definitions from 51 trials: 54 (14%) outcomes had insufficient definitions in the public trial registry to allow comparison between what was reported in the methods section of articles and what had been registered; 116 (30%) were similar in the two sources; 11 (3%) had been modified in the published article (e.g., with a different threshold); and 204 (53%) had not been prespecified in registries. Examples are provided in Table 4. Primary outcomes were often prespecified (even if description was not clear enough to enable comparison) (42/64, 66%).

**Table 4:** Prespecification of outcomes measured with BMDs in the included trials (*n* = 464 outcomes). For each included trial, we looked for the corresponding entry in a public clinical trial registry (e.g., ClinicalTrials.gov) by looking for the trial registration number reported in the articles on January 2020 (and June 2020 for articles added during peer-review)

Comparison between outcome definitions in published articles and entries in public trial registries	Number of outcomes (%)	Example
Impossible because the trial is not registered*	79 (17)	–
Outcome definition is not clear enough for comparison	54 (14)**	In the trial [22]: Published outcome: “MVPA was calculated using 3 axes based on 60 s epochs. Freedson-VM cut-off points were used to distinguish between light, moderate and vigorous PA.” Outcome registered: “Objective physical activity (accelerometer data)”
Similar outcome definitions reported in the 2 sources	116 (30)**	In the trial [23]: Published outcomes: “time with glucose concentration […] hyperglycemic (> 10.0 mmol/L and > 16.7 mmol/L)” Outcomes registered: “Time spent above target glucose (10.0 mmol/l) (180 mg/dl) [Time Frame: 12-week intervention phase]” and “The time with glucose levels in the significant hyperglycemia (glucose levels > 16.7 mmol/l) (300 mg/dl) [Time Frame: 12 week intervention phase]”
Outcome definition was modified in the published article	11 (3)**	In the trial [24], time frames during which BMD outcome data analyzed were modified; published outcome: “M-value (24 h […], 08.00–12.00 h […], 12.00–24.00 h […], 00.00–06.00 h […])” Outcomes registered: “M-value (24:00, 8:00–12:00, 12:00–24:00, 0:00–8:00)”
Outcome in the published article was not registered	204 (53)**	Among outcomes registered for the trial [25], only “% time within target range (3.9–10 mmol/L)” and “Incidence rate of hypoglycemic events” were registered. Published article also reported: “% time > 10 mmol/L”; “% time > 15 mmol/L” “% time > 20 mmol/L” and “Mean (SD) glucose values”

*Including one trial for which the registration number provided in the published article linked to a different study

**Denominator is 385 outcomes for which comparison was possible (464–79)

### Management of missing and incomplete BMD outcome data in RCTs

Regarding missing BMD outcome data, 26 (35%) trials did not report enough information to understand how missing outcome data were managed, and 25 (33%) simply excluded patients with missing outcome data. The remaining trials used classical methods to deal with missing BMD outcome data (e.g., multiple imputation [*n* = 8], use of models robust for missing data [*n* = 7], last observation carried forward [*n* = 2], missing data inferred by investigators [*n* = 2], missing data considered as failures [*n* = 2]).

Regarding incomplete BMD outcome data, 51 (68%) trials did not mention this issue. For the 24 (32%) remaining trials, incomplete BMD outcome data were neglected until it was greater than a given threshold. For example, in a trial of knee osteoarthritis using an accelerometer, “Data were eligible if patients wore the meter ≥3 days [over 5 days], for ≥ 8 h per day” [26]. In another trial of diabetes using a continuous glucose monitoring system, an additional analysis was restricted to “participants with sensor glucose data availability for at least 50% of the time over the 12-week study period” [23]. Similar to outcome definitions, the threshold for incomplete outcome data varied in all trials. The number of patients with levels of incomplete BMD outcome data over the threshold was reported in 16 (21%) articles. This could represent up to 27% of the number of randomized patients [23].

## Discussion

To our knowledge, this is the first methodologic systematic review describing how BMDs are used to assess outcomes in a broad sample of recent RCTs, across different conditions. Our findings showed that many trials, including drug development trials, are using BMDs to collect both primary and secondary outcome measures.

Because of no validated search strategy to retrieve trials using BMDs to collect outcomes (with ever-changing names used in the literature: sensors, wearables, trackers, continuous monitoring devices, etc.), we may have missed some trials. To account for this issue, we complemented our electronic search strategy with a page-by-page hand search in the top 5 general medical journals. This ensured that the most influential trials, likely to impact practice, would be included, but may have led to omit some trials published in specialized journals in digital health.

In this sample of trials, we highlighted several challenges that may affect the validity and transferability of results from trials using BMDs to collect outcomes. First, our review highlighted an alarming heterogeneity in the definition of outcomes measuring the same concept of interest across the RCTs. With this heterogeneity, comparing and combining RCT results (e.g., in systematic reviews and clinical guidelines) could be difficult [27]. One solution to improve the harmonization of outcomes in trials for a given condition is the definition of core outcome sets (COSs). COSs are agreed-upon standardized sets of outcomes that should be minimally measured in all trials of a specific clinical area [28]. To our knowledge, no existing COS promotes the use of outcomes measured by BMDs nor proposes solutions for the standardization of these measures. For example, a recent COS proposed for type 1 diabetes includes only glycated hemoglobin as measure of glycemic control, whereas most recent major trials of this disease have used results from continuous glucose monitoring [23, 29, 30]. Therefore, we must develop or update existing COSs to include outcomes measured using BMDs [31].

Second, BMDs are transforming outcome assessment in clinical trials: instead of single measures at given time points, researchers can now analyze dense longitudinal data and better understand the dynamic effects of interventions over time and outside of usual experimental contexts. Still, at the trial level, this situation contributes to an excessive number of summarized measures of the treatment effect generated from the same raw data, with often no pre-established priority. For example, in one trial comparing high-dose metformin to a low-dose metformin/linagliptin combination to improve glycemic variability in type 2 diabetes, the investigators measured up to 25 different outcomes using the data from the continuous glucose monitoring system (24-h mean glucose level; night-time mean glucose level; mean amplitude of glycemic excursions; mean preprandial glucose levels before breakfast, lunch and dinner; mean postprandial glucose levels after breakfast, lunch and dinner, etc.) [21]. Interpreting results was difficult, especially for outcomes with discordant directions of results. This situation represents a risk of selective outcome reporting bias. Our review shows that, in trials using BMDs to collect outcomes, 50% of analyses were not prespecified. This is twice what was known for trials not using BMDs [32–34]. Furthermore, in 14% of trials included in this study, outcome definitions reported in public trial registries were insufficient to allow comparison (e.g., outcomes reported only as “continuous glucose monitoring values” or “accelerometer data” without further details). To account for this problem, we call for extensions to the SPIRIT and CONSORT statements specific to the inclusion of BMD outcome data in clinical trial protocols and reports, similar to what has been done for the integration of patient-reported outcome data in these documents [35–37].

Third, the need to ensure that measurements from BMDs are valid, reliable, and responsive has been underlined in multiple publications and guidelines [4, 10, 13, 38]. Yet, our study shows that only 26% of studies did report enough information to allow readers to easily assess these items. Retrieving the studies evaluating the measurement properties of the devices used in trials is a difficult task.

Finally, our review uncovers the problem posed by incomplete outcome data due to poor compliance with the BMD during the trial. This is a new problem in trial methodology, different from the management of missing outcome data. Conventional data imputation strategies are inadequate in capturing temporal variations in multivariate time series and therefore are not recommended [16]. We lack consensus on how to deal with incomplete outcome data [20]. In our review, about 70% of trials did not report any information on this issue. This situation may have a considerable impact on results; indeed, a recent study showed that compliance with BMDs is often low [39]. A remaining question is how an imbalance in a trial in terms of compliance with BMD may affect the estimates of treatment effect.

All problems investigated (heterogeneity of outcome definitions, management of incomplete data, poor reporting of metrologic properties of sensors) affected all studies, including those using BMDs to measure their primary outcomes.

Our study has some limitations. First, as stated previously, our search strategy may not have identified all available studies because of no validated search strategy to retrieve trials using BMDs as outcomes. Second, we only considered 1 year of clinical trials. However, including older studies would not likely have changed our findings on how key methodological challenges are addressed in RCTs using BMDs to measure outcomes.

## Conclusion

The integration of real-world data obtained from BMDs in trials has the power to fundamentally change how clinical trials are designed and conducted. To make the most of this revolution, trialists must account for the methodological specificities of these tools. Especially, precise reporting of outcome definitions used in trials (including how incomplete data are handled) and how these outcome definitions were prespecified will strengthen the validity and transferability of results. In parallel, companies, researchers, initiatives to improve the quality of trials (e.g., EQUATOR, COMET) and regulation authorities need to work on standards to summarize data from BMDs in clinical research.

## Supplementary information

**Additional file 1: Appendix 1:** Search strategy. **Appendix 2:** List of studies included in the review (*n* = 75). **Appendix 3:** Outcome definitions used to assess a given concept of interest (n = 75 trials measuring 464 outcomes). The Jaccard similarity coefficient measures the similarity of outcomes used in 2 trials. It ranges from 0 (no overlap of outcomes) to 1 (complete overlap of outcomes). The Jaccard similarity coefficient is calculated as s/(u1 + u2 + s), where s is the number of similar outcome definitions the 2 trials share, and u1 and u2 are the number of outcome definitions that are unique to each of the trials. The average Jaccard similarity coefficient is the arithmetic mean of coefficients for all possible combinations of 2 trials. **Appendix 4:** Outcome definitions in randomized controlled trials that used biometric monitoring devices (BMDs) for adherence to treatment, blood pressure control, diabetic foot assessment, heart rate variability and sleep quality. Each node represents a given outcome definition characterized by its domain, measurement method, metric, aggregation method and time frame. The size of nodes represents the number of times each outcome definition was used in the included trials. Outcome definitions are clustered by outcome domains. **Appendix 5:** List of unique definitions of outcomes measured with BMDs.

## Data Availability

The datasets used and/or analyzed during the current study are available from the corresponding author on reasonable request.
