# Supplementary material for: Use of wearable biometric monitoring devices to measure outcomes in randomized clinical trials: a methodological systematic review
Source: BMC Med. 2020 Nov 6;18:310. doi: 10.1186/s12916-020-01773-w (PMC7646072; doi:10.1186/s12916-020-01773-w)
Supplement: Supplementary file 1 — Additional file 1: Appendix 1: Search strategy. Appendix 2: List of studies included in the review (n = 75). Appendix 3: Outcome definitions used to assess a given concept of interest (n = 75 trials measuring 464 outcomes). The Jaccard similarity coefficient measures the similarity of outcomes used in 2 trials. It ranges from 0 (no overlap of outcomes) to 1 (complete overlap of outcomes). The Jaccard similarity coefficient is calculated as s/(u1 + u2 + s), where s is the number of similar outcome definitions the 2 trials share, and u1 and u2 are the number of outcome definitions that are unique to each of the trials. The average Jaccard similarity coefficient is the arithmetic mean of coefficients for all possible combinations of 2 trials. Appendix 4: Outcome definitions in randomized controlled trials that used biometric monitoring devices (BMDs) for adherence to treatment, blood pressure control, diabetic foot assessment, heart rate variability and sleep quality. Each node represents a given outcome definition characterized by its domain, measurement method, metric, aggregation method and time frame. The size of nodes represents the number of times each outcome definition was used in the included trials. Outcome definitions are clustered by outcome domains. Appendix 5: List of unique definitions of outcomes measured with BMDs. [file 12916_2020_1773_MOESM1_ESM.docx]

**Appendix 1a: Search strategy for PubMed (February 2019)**

| #1 | Biometric monitoring devices | continuous[Title/Abstract]  OR wearable[Title/Abstract]  OR device*[Title/Abstract]  OR closed-loop[Title/Abstract]  OR instrumentation [MeSH] |
| --- | --- | --- |
| #2 | Study type | Randomly [Title/Abstract]  OR trial [Title/Abstract]  OR clinical trials as topic[mesh:noexp]  OR placebo [Title/Abstract]  OR randomized [Title/Abstract]  OR randomized [Title/Abstract]  OR controlled clinical trial [Publication Type]  OR randomized controlled trial [Publication Type]  NOT (animals[MeSH] NOT humans[MeSH] |
| #3 | Population | NOT child* |
| #4 | Exclusion | NOT Surgery [Title/Abstract]  NOT Intensive Care [Title/Abstract] |
| #6 | #1 AND #2 AND #3 AND #4 |  |

**Appendix 1b: Keywords and MeSH terms added to the search strategy during peer-review (June 2020)**

| #1 | Biometric monitoring devices | accelerometry [MeSH]  OR smartphone [MeSH]  OR wearable device [MeSH]  OR mobile applications [MeSH] |
| --- | --- | --- |

**Appendix 2: List of studies included in the review (n=75)**

1. Afzelius P, Molsted S, Tarnow L: **Intermittent vacuum treatment with VacuMed does not improve peripheral artery disease or walking capacity in patients with intermittent claudication**. *Scandinavian Journal of Clinical and Laboratory Investigation* 2018, **78**(6):456-463.

2. Arbillaga-Etxarri A, Gimeno-Santos E, Barberan-Garcia A, Balcells E, Benet M, Borrell E, Celorrio N, Delgado A, Jané C, Marin A *et al*: **Long-term efficacy and effectiveness of a behavioural and community-based exercise intervention (Urban Training) to increase physical activity in patients with COPD: a randomised controlled trial**. *The European respiratory journal* 2018, **52**(4).

3. Bally L, Thabit H, Hartnell S, Andereggen E, Ruan Y, Wilinska ME, Evans ML, Wertli MM, Coll AP, Stettler C *et al*: **Closed-Loop Insulin Delivery for Glycemic Control in Noncritical Care**. *New England journal of medicine* 2018, **379**(6):547‐556.

4. Banhiran W, Assanasen P, Nopmaneejumrudlers C, Nujchanart N, Srechareon W, Chongkolwatana C, Metheetrairut C: **Adjustable thermoplastic oral appliance versus positive airway pressure for obstructive sleep apnea**. *Laryngoscope* 2018, **128**(2):516-522.

5. Blough J, Loprinzi PD: **Experimentally investigating the joint effects of physical activity and sedentary behavior on depression and anxiety: A randomized controlled trial**. *Journal of affective disorders* 2018, **239**((Blough J.; Loprinzi P.D., pdloprin@olemiss.edu) Physical Activity Epidemiology Laboratory, Exercise Psychology Laboratory, Department of Health, Exercise Science and Recreation Management, The University of Mississippi, , MS, United States):258-268.

6. Borlaug BA, Anstrom KJ, Lewis GD, Shah SJ, Levine JA, Koepp GA, Givertz MM, Felker GM, LeWinter MM, Mann DL *et al*: **Effect of Inorganic Nitrite vs Placebo on Exercise Capacity Among Patients With Heart Failure With Preserved Ejection Fraction: the INDIE-HFpEF Randomized Clinical Trial**. *Jama* 2018, **320**(17):1764‐1773.

7. Bus SA, van Netten JJ, Kottink AIR, Manning EA, Spraul M, Woittiez AJ, van Baal JG: **The efficacy of removable devices to offload and heal neuropathic plantar forefoot ulcers in people with diabetes: a single-blinded multicentre randomised controlled trial**. *International wound journal* 2018, **15**(1):65‐74.

8. Carter SJ, Hunter GR, Norian LA, Turan B, Rogers LQ: **Ease of walking associates with greater free-living physical activity and reduced depressive symptomology in breast cancer survivors: pilot randomized trial**. *Supportive care in cancer : official journal of the Multinational Association of Supportive Care in Cancer* 2018, **26**(5):1675-1683.

9. Chaoul A, Milbury K, Spelman A, Basen-Engquist K, Hall MH, Wei Q, Shih YT, Arun B, Valero V, Perkins GH *et al*: **Randomized trial of Tibetan yoga in patients with breast cancer undergoing chemotherapy**. *Cancer* 2018, **124**(1):36-45.

10. Chlup R, Runzis S, Castaneda J, Lee SW, Nguyen X, Cohen O: **Complex Assessment of Metabolic Effectiveness of Insulin Pump Therapy in Patients with Type 2 Diabetes beyond HbA1c Reduction**. *Diabetes Technology and Therapeutics* 2018, **20**(2):153-159.

11. Cho JH, Lee JY, Lee S, Park H, Choi SW, Kim JC: **Effect of intradialytic exercise on daily physical activity and sleep quality in maintenance hemodialysis patients**. *International Urology and Nephrology* 2018, **50**(4):745-754.

12. Chokshi NP, Adusumalli S, Small DS, Morris A, Feingold J, Ha YP, Lynch MD, Rareshide CAL, Hilbert V, Patel MS: **Loss-framed financial incentives and personalized goal-setting to increase physical activity among ischemic heart disease patients using wearable devices: the ACTIVE REWARD randomized trial**. *Journal of the american heart association* 2018, **7**(12).

13. Davtyan K, Shatakhtsyan V, Poghosyan H, Deev A, Tarasov A, Kharlap M, Serdyuk S, Simonyan G, Boytcov S: **Radiofrequency versus Cryoballoon Ablation of Atrial Fibrillation: An Evaluation Using ECG, Holter Monitoring, and Implantable Loop Recorders to Monitor Absolute and Clinical Effectiveness**. *Biomed Res Int* 2018, **2018**:3629384.

14. Desteghe L, Vijgen J, Koopman P, D DI-B, Schurmans J, Dendale P, Heidbuchel H: **Telemonitoring-based feedback improves adherence to non-Vitamin K antagonist oral anticoagulants intake in patients with atrial fibrillation**. *European heart journal* 2018, **39**(16):1394-1403.

15. Dunn SL, Robbins LB, Smith SW, Ranganathan R, DeVon HA, Collins EG, Hong HG, Tintle NL: **Enhancing physical activity in cardiac patients who report hopelessness: Feasibility testing of an intervention**. *Health Education Journal* 2018.

16. Duscha BD, Piner LW, Patel MP, Craig KP, Brady M, McGarrah RW, Chen C, Kraus WE: **Effects of a 12-week mHealth program on peak VO2 and physical activity patterns after completing cardiac rehabilitation: a randomized controlled trial**. *American heart journal* 2018, **199**:105‐114.

17. Ebbeling CB, Feldman HA, Klein GL, Wong JMW, Bielak L, Steltz SK, Luoto PK, Wolfe RR, Wong WW, Ludwig DS: **Effects of a low carbohydrate diet on energy expenditure during weight loss maintenance: randomized trial**. *BMJ (Clinical research ed)* 2018, **363**:k4583.

18. Foltynski P, Ladyzynski P, Pankowska E, Mazurczak K: **Efficacy of automatic bolus calculator with automatic speech recognition in patients with type 1 diabetes: a randomized cross-over trial**. *Journal of diabetes* 2018, **10**(7):600‐608.

19. Gerards MC, Venema GE, Patberg KW, Kross M, Potter van Loon BJ, Hageman IMG, Snijders D, Brandjes DPM, Hoekstra JBL, Vriesendorp TM *et al*: **Dapagliflozin for prednisone-induced hyperglycaemia in acute exacerbation of chronic obstructive pulmonary disease**. *Diabetes, obesity & metabolism* 2018, **20**(5):1306‐1310.

20. Golsteijn RHJ, Bolman C, Volders E, Peels DA, De Vries H, Lechner L: **Short-term efficacy of a computer-tailored physical activity intervention for prostate and colorectal cancer patients and survivors: A randomized controlled trial**. *International journal of behavioral nutrition and physical activity* 2018, **15**(1).

21. Heinemann L, Freckmann G, Ehrmann D, Faber-Heinemann G, Guerra S, Waldenmaier D, Hermanns N: **Real-time continuous glucose monitoring in adults with type 1 diabetes and impaired hypoglycaemia awareness or severe hypoglycaemia treated with multiple daily insulin injections (HypoDE): a multicentre, randomised controlled trial**. *Lancet (London, England)* 2018, **391**(10128):1367-1377.

22. Howsmon DP, Baysal N, Buckingham BA, Forlenza GP, Ly TT, Maahs DM, Marcal T, Towers L, Mauritzen E, Deshpande S *et al*: **Real-Time Detection of Infusion Site Failures in a Closed-Loop Artificial Pancreas**. *Journal of diabetes science and technology* 2018.

23. Imai S, Kajiyama S, Hashimoto Y, Nitta A, Miyawaki T, Matsumoto S, Ozasa N, Tanaka M, Fukui M: **Consuming snacks mid-afternoon compared with just after lunch improves mean amplitude of glycaemic excursions in patients with type 2 diabetes: A randomized crossover clinical trial**. *Diabetes Metab* 2018, **44**(6):482-487.

24. Jolly K, Sidhu MS, Hewitt CA, Coventry PA, Daley A, Jordan R, Heneghan C, Singh S, Ives N, Adab P *et al*: **Self management of patients with mild COPD in primary care: randomised controlled trial**. *BMJ (Clinical research ed)* 2018, **361**:k2241.

25. Kaplan KA, Talavera DC, Harvey AG: **Rise and shine: A treatment experiment testing a morning routine to decrease subjective sleep inertia in insomnia and bipolar disorder**. *Behaviour research and therapy* 2018, **111**:106-112.

26. Katz P, Margaretten M, Gregorich S, Trupin L: **Physical Activity to Reduce Fatigue in Rheumatoid Arthritis: A Randomized Controlled Trial**. *Arthritis Care Res (Hoboken)* 2018, **70**(1):1-10.

27. Keukenkamp R, Merkx MJ, Busch-Westbroek TE, Bus SA: **An Explorative Study on the Efficacy and Feasibility of the Use of Motivational Interviewing to Improve Footwear Adherence in Persons with Diabetes at High Risk for Foot Ulceration**. *Journal of the American Podiatric Medical Association* 2018, **108**(2):90-99.

28. Kim JD, Park CY, Cha BY, Ahn KJ, Kim IJ, Park KS, Lee HW, Min KW, Won JC, Chung MY *et al*: **Comparison of Adherence to Glimepiride/Metformin Sustained Release Once-daily Versus Glimepiride/Metformin Immediate Release BID Fixed-combination Therapy Using the Medication Event Monitoring System in Patients With Type 2 Diabetes**. *Clinical Therapeutics* 2018, **40**(5):752-761.e752.

29. Kim WS, Cho S, Park SH, Lee JY, Kwon S, Paik NJ: **A low cost kinect-based virtual rehabilitation system for inpatient rehabilitation of the upper limb in patients with subacute stroke**. *Medicine (United States)* 2018, **97**(25).

30. Kloek CJJ, van Dongen JM, de Bakker DH, Bossen D, Dekker J, Veenhof C: **Cost-effectiveness of a blended physiotherapy intervention compared to usual physiotherapy in patients with hip and/or knee osteoarthritis: a cluster randomized controlled trial**. *BMC Public Health* 2018, **18**(1):1082.

31. Levitt DL, Spanakis EK, Ryan KA, Silver KD: **Insulin Pump and Continuous Glucose Monitor Initiation in Hospitalized Patients with Type 2 Diabetes Mellitus**. *Diabetes Technol Ther* 2018, **20**(1):32-38.

32. Little SA, Speight J, Leelarathna L, Walkinshaw E, Tan HK, Bowes A, Lubina-Solomon A, Chadwick TJ, Stocken DD, Brennand C *et al*: **Sustained reduction in severe hypoglycemia in adults with type 1 diabetes complicated by impaired awareness of hypoglycemia: Two-year follow-up in the HypoCOMPaSS randomized clinical Trial**. *Diabetes Care* 2018, **41**(8):1600-1607.

33. Luo Y, Wang XQ, Ni WJ, Ding B, Xu XH, Ye L, Ma JH, Zhu J: **Comparison of Efficacy and Economic Value of Prandilin 25 and Humalog Mix 25 in Patients with Newly Diagnosed Type 2 Diabetes by a Continuous Glucose Monitoring System**. *Diabetes Ther* 2018, **9**(6):2219-2228.

34. Mabweazara SZ, Leach LL, Ley C, Smith M: **A six week contextualised physical activity intervention for women living with HIV and AIDS of low socioeconomic status: a pilot study**. *AIDS Care–Psychological and Socio-Medical Aspects of AIDS/HIV* 2018, **30**((Mabweazara S.Z., smabweazara@gmail.com; Leach L.L.) Department of Sport, Recreation and Exercise Science, University of the Western Cape, Cape Town, South Africa):61-65.

35. Manzur F, Rico A, Romero JD, Rodriguez-Martinez CE: **Efficacy and Safety of Valsartan or Chlorthalidone vs. Combined Valsartan and Chlorthalidone in Patients With Mild to Moderate Hypertension: The VACLOR Study**. *Clinical Medicine Insights: Cardiology* 2018, **12**((Manzur F.) School of Medicine, Universidad de Cartagena, Cartagena, Colombia).

36. Márquez Contreras E, Márquez Rivero S, Rodríguez García E, López-García-Ramos L, Carlos Pastoriza Vilas J, Baldonedo Suárez A, Gracia Diez C, Gil Guillén V, Martell Claros N: **Specific hypertension smartphone application to improve medication adherence in hypertension: a cluster-randomized trial**. *Current medical research and opinion* 2019, **35**(1):167-173.

37. McDermott MM, Spring B, Berger JS, Treat-Jacobson D, Conte MS, Creager MA, Criqui MH, Ferrucci L, Gornik HL, Guralnik JM *et al*: **Effect of a Home-Based Exercise Intervention of Wearable Technology and Telephone Coaching on Walking Performance in Peripheral Artery Disease: the HONOR Randomized Clinical Trial**. *Jama* 2018, **319**(16):1665‐1676.

38. McGibbon CA, Sexton A, Jayaraman A, Deems-Dluhy S, Gryfe P, Novak A, Dutta T, Fabara E, Adans-Dester C, Bonato P: **Evaluation of the Keeogo exoskeleton for assisting ambulatory activities in people with multiple sclerosis: an open-label, randomized, cross-over trial**. *J Neuroeng Rehabil* 2018, **15**(1):117.

39. Meester D, Al-Yahya E, Dennis A, Collett J, Wade DT, Ovington M, Liu F, Meaney A, Cockburn J, Johansen-Berg H *et al*: **A randomized controlled trial of a walking training with simultaneous cognitive demand (dual-task) in chronic stroke**. *European journal of neurology* 2018((Meester D., d.meester@alumni.maastrichtuniversity.nl; Al-Yahya E.; Collett J.; Wade D.T.; Ovington M.; Liu F.; Meaney A.; Dawes H.) Movement Science Group, School of Life Sciences, Oxford Brookes University, Oxford, United Kingdom).

40. Moore DJ, Pasipanodya EC, Umlauf A, Rooney AS, Gouaux B, Depp CA, Atkinson JH, Montoya JL: **Individualized texting for adherence building (iTAB) for methamphetamine users living with HIV: A pilot randomized clinical trial**. *Drug and Alcohol Dependence* 2018, **189**((Moore D.J., djmoore@ucsd.edu; Pasipanodya E.C.; Umlauf A.; Rooney A.S.; Gouaux B.; Depp C.A.; Atkinson J.H.; Montoya J.L.) Department of Psychiatry, University of California, School of Medicine, La Jolla, San Diego, CA, United States):154-160.

41. Moscardó V, Bondia J, Ampudia-Blasco FJ, Fanelli CG, Lucidi P, Rossetti P: **Plasma Insulin Levels and Hypoglycemia Affect Subcutaneous Interstitial Glucose Concentration**. *Diabetes Technology and Therapeutics* 2018, **20**(4):263-273.

42. Nawrat-Szoltysik AJ, Polak A, Malecki A, Piejko L, Grzybowska-Ganszczyk D, Krecichwost M, Opara J: **Effect of physical activity on the sequelae of osteoporosis in female residents of residential care facilities**. *Advances in Clinical and Experimental Medicine* 2018, **27**(5):633-642.

43. Okajima F, Nakamura Y, Yamaguchi Y, Shuto Y, Kato K, Sugihara H, Emoto N: **Basal–Bolus Insulin Therapy with Gla-300 During Hospitalization Reduces Nocturnal Hypoglycemia in Patients with Type 2 Diabetes Mellitus: A Randomized Controlled Study**. *Diabetes Therapy* 2018, **9**(3):1049-1059.

44. O'Neill B, O'Shea O, McDonough S, McGarvey L, Bradbury I, Arden M, Troosters T, Cosgrove D, McManus T, McDonnell T *et al*: **Clinician-Facilitated Physical Activity Intervention Versus Pulmonary Rehabilitation for Improving Physical Activity in COPD: A Feasibility Study**. *Copd* 2018, **15**(3):254-264.

45. Oskarsson P, Antuna R, Geelhoed-Duijvestijn P, Krger J, Weitgasser R, Bolinder J: **Impact of flash glucose monitoring on hypoglycaemia in adults with type 1 diabetes managed with multiple daily injection therapy: a pre-specified subgroup analysis of the IMPACT randomised controlled trial**. *Diabetologia* 2018, **61**(3):539-550.

46. Pettus J, Reeds D, Cavaiola TS, Boeder S, Levin M, Tobin G, Cava E, Thai D, Shi J, Yan H *et al*: **Effect of a glucagon receptor antibody (REMD-477) in type 1 diabetes: A randomized controlled trial**. *Diabetes, Obesity and Metabolism* 2018, **20**(5):1302-1305.

47. Petzold MB, Mumm JLM, Bischoff S, Große J, Plag J, Brand R, Ströhle A: **Increasing physical activity and healthy diet in outpatients with mental disorders: a randomized-controlled evaluation of two psychological interventions**. *European archives of psychiatry and clinical neuroscience* 2019, **269**(5):529-542.

48. Polgreen LA, Anthony C, Carr L, Simmering JE, Evans NJ, Foster ED, Segre AM, Cremer JF, Polgreen PM: **The effect of automated text messaging and goal setting on pedometer adherence and physical activity in patients with diabetes: A randomized controlled trial**. *Plos one* 2018, **13**(5):e0195797.

49. Poulter NR, Savopoulos C, Anjum A, Apostolopoulou M, Chapman N, Cross M, Falaschetti E, Fotiadis S, James RM, Kanellos I *et al*: **Randomized Crossover Trial of the Impact of Morning or Evening Dosing of Antihypertensive Agents on 24-Hour Ambulatory Blood Pressure**. *Hypertension (Dallas, Tex : 1979)* 2018, **72**(4):870-873.

50. Prince SA, Reed JL, Cotie LM, Harris J, Pipe AL, Reid RD: **Results of the Sedentary Intervention Trial in Cardiac Rehabilitation (SIT-CR Study): A pilot randomized controlled trial**. *International journal of cardiology* 2018, **269**((Prince S.A., sprinceware@ottawaheart.ca; Reed J.L.; Cotie L.M.; Harris J.; Pipe A.L.; Reid R.D.) Division of Cardiac Prevention and Rehabilitation, University of Ottawa Heart Institute, Canada):317-324.

51. Rantanen JM, Riahi S, Johansen MB, Schmidt EB, Christensen JH: **Effects of marine n-3 polyunsaturated fatty acids on heart rate variability and heart rate in patients on chronic dialysis: A randomized controlled trial**. *Nutrients* 2018, **10**(9).

52. Riddle MC, Nahra R, Han J, Castle J, Hanavan K, Hompesch M, Huffman D, Strange P, Ohman P: **Control of Postprandial Hyperglycemia in Type 1 Diabetes by 24-Hour Fixed-Dose Coadministration of Pramlintide and Regular Human Insulin: A Randomized, Two-Way Crossover Study**. *Diabetes Care* 2018, **41**(11):2346-2352.

53. Rienstra M, Hobbelt AH, Alings M, Tijssen JGP, Smit MD, Brügemann J, Geelhoed B, Tieleman RG, Hillege HL, Tukkie R *et al*: **Targeted therapy of underlying conditions improves sinus rhythm maintenance in patients with persistent atrial fibrillation: Results of the RACE 3 trial**. *European heart journal* 2018, **39**(32):2987-2996.

54. Rohla M: **Hypertension News-Screen: Spironolactone versus clonidine as a fourth-drug therapy for resistant hypertension–The ReHOT randomized study (resistant hypertension optimal treatment)**. *Journal fur Hypertonie* 2018, **22**(1):21-22.

55. Rohla M, Tscharre M, Huber K, Weiss TW: **Lowering blood pressure in primary care in Vienna (LOW-BP-VIENNA): A cluster-randomized trial**. *Wiener klinische wochenschrift* 2018, **130**(23-24):698-706.

56. Shim CY, Kim D, Park S, Lee CJ, Cho HJ, Ha JW, Cho YJ, Hong GR: **Effects of continuous positive airway pressure therapy on left ventricular diastolic function: A randomised, shamcontrolled clinical trial**. *European Respiratory Journal* 2018, **51**(2).

57. Sinharay R, Gong J, Barratt B, Ohman-Strickland P, Ernst S, Kelly FJ, Zhang JJ, Collins P, Cullinan P, Chung KF: **Respiratory and cardiovascular responses to walking down a traffic-polluted road compared with walking in a traffic-free area in participants aged 60 years and older with chronic lung or heart disease and age-matched healthy controls: a randomised, crossover study**. *Lancet (London, England)* 2018, **391**(10118):339-349.

58. Sletten TL, Magee M, Murray JM, Gordon CJ, Lovato N, Kennaway DJ, Gwini SM, Bartlett DJ, Lockley SW, Lack LC *et al*: **Efficacy of melatonin with behavioural sleep-wake scheduling for delayed sleep-wake phase disorder: A double-blind, randomised clinical trial**. *PLoS medicine* 2018, **15**(6):e1002587.

59. Steinhubl SR, Waalen J, Edwards AM, Ariniello LM, Mehta RR, Ebner GS, Carter C, Baca-Motes K, Felicione E, Sarich T *et al*: **Effect of a Home-Based Wearable Continuous ECG Monitoring Patch on Detection of Undiagnosed Atrial Fibrillation: The mSToPS Randomized Clinical Trial**. *Jama* 2018, **320**(2):146-155.

60. Suzuki R, Eiki JI, Moritoyo T, Furihata K, Wakana A, Ohta Y, Tokita S, Kadowaki T: **Effect of short-term treatment with sitagliptin or glibenclamide on daily glucose fluctuation in drug-naïve Japanese patients with type 2 diabetes mellitus**. *Diabetes, Obesity and Metabolism* 2018, **20**(9):2274-2281.

61. Svendsen MT, Andersen F, Andersen KH, Pottegård A, Johannessen H, Möller S, August B, Feldman SR, Andersen KE: **A smartphone application supporting patients with psoriasis improves adherence to topical treatment: a randomized controlled trial**. *British journal of dermatology* 2018, **179**(5):1062-1071.

62. Takahashi H, Nishimura R, Tsujino D, Utsunomiya K: **Which is better, high-dose metformin monotherapy or low-dose metformin/linagliptin combination therapy, in improving glycemic variability in type 2 diabetes patients with insufficient glycemic control despite low-dose metformin monotherapy? A randomized, cross-over, continuous glucose monitoring-based pilot study**. *Journal of diabetes investigation* 2018((Takahashi H., hiro6117@jikei.ac.jp; Nishimura R.; Tsujino D.; Utsunomiya K.) Division of Diabetes, Metabolism and Endocrinology, Department of Internal Medicine, Jikei University School of Medicine, Tokyo, Japan).

63. Takeishi S, Tsuboi H, Takekoshi S: **Comparison of morning basal + 1 bolus insulin therapy (insulin glulisine + insulin glargine 300 U/mL vs insulin lispro + insulin glargine biosimilar) using continuous glucose monitoring: a randomized crossover study**. *Journal of diabetes investigation* 2018, **9**(1):91‐99.

64. Tauschmann M, Thabit H, Bally L, Allen JM, Hartnell S, Wilinska ME, Ruan Y, Sibayan J, Kollman C, Cheng P *et al*: **Closed-loop insulin delivery in suboptimally controlled type 1 diabetes: a multicentre, 12-week randomised trial**. *Lancet (London, England)* 2018, **392**(10155):1321-1329.

65. ter Hoeve N, Sunamura M, Stam HJ, Boersma E, Geleijnse ML, van Domburg RT, van den Berg-Emons RJG: **Effects of two behavioral cardiac rehabilitation interventions on physical activity: A randomized controlled trial**. *International journal of cardiology* 2018, **255**((ter Hoeve N., n.terhoeve@erasmusmc.nl; Sunamura M.) Capri Cardiac Rehabilitation, Rotterdam, Netherlands):221-228.

66. Troosters T, Maltais F, Leidy N, Lavoie KL, Sedeno M, Janssens W, Garcia-Aymerich J, Erzen D, De Sousa D, Korducki L *et al*: **Effect of Bronchodilation, Exercise Training, and Behavior Modification on Symptoms and Physical Activity in Chronic Obstructive Pulmonary Disease**. *American journal of respiratory and critical care medicine* 2018, **198**(8):1021-1032.

67. Tsujino D, Nishimura R, Onda Y, Seo C, Ando K, Utsunomiya K: **Biphasic insulin aspart-30 reduces glycemic variability to a greater degree than insulin detemir: A randomized controlled trial of once-daily insulin regimens using continuous glucose monitoring**. *Journal of diabetes investigation* 2018, **9**(3):573-578.

68. Uhl JF, Benigni JP, Chahim M, Fréderic D: **Prospective randomized controlled study of patient compliance in using a compression stocking: Importance of recommendations of the practitioner as a factor for better compliance**. *Phlebology* 2018, **33**(1):36-43.

69. Varas AB, Córdoba S, Rodríguez-Andonaegui I, Rueda MR, García-Juez S, Vilaró J: **Effectiveness of a community-based exercise training programme to increase physical activity level in patients with chronic obstructive pulmonary disease: A randomized controlled trial**. *Physiotherapy research international : the journal for researchers and clinicians in physical therapy* 2018, **23**(4):e1740.

70. Villaron C, Cury F, Eisinger F, Cappiello MA, Marqueste T: **Telehealth applied to physical activity during cancer treatment: a feasibility, acceptability, and randomized pilot study**. *Supportive care in cancer* 2018, **26**(10):3413‐3421.

71. Widyastuti K, Makhabah DN, Setijadi AR, Sutanto YS, Suradi, Ambrosino N: **Benefits and costs of home pedometer assisted physical activity in patients with COPD. A preliminary randomized controlled trial**. *Pulmonology* 2018, **24**(4):211-218.

72. Winding KM, Munch GW, Iepsen UW, Van Hall G, Pedersen BK, Mortensen SP: **The effect on glycaemic control of low-volume high-intensity interval training versus endurance training in individuals with type 2 diabetes**. *Diabetes Obes Metab* 2018, **20**(5):1131-1139.

73. Wouda MF, Lundgaard E, Becker F, Strøm V: **Effects of moderate- and high-intensity aerobic training program in ambulatory subjects with incomplete spinal cord injury-a randomized controlled trial**. *Spinal cord* 2018, **56**(10):955-963.

74. Yeung WF, Lai AY, Ho FY, Suen LK, Chung KF, Ho JY, Ho LM, Yu BY, Chan LY, Lam TH: **Effects of Zero-time Exercise on inactive adults with insomnia disorder: a pilot randomized controlled trial**. *Sleep medicine* 2018, **52**:118-127.

75. Zhang YP, Liao WJ, Xia WG: **Effect of Acupuncture Cooperated with Low-frequency Repetitive Transcranial Magnetic Stimulation on Chronic Insomnia: A Randomized Clinical Trial**. *Current medical science* 2018, **38**(3):491-498.

**Appendix 3. Outcome definitions used to assess a given concept of interest (n=75 trials measuring 464 outcomes).** The Jaccard similarity coefficient measures the similarity of outcomes used in 2 trials. It ranges from 0 (no overlap of outcomes) to 1 (complete overlap of outcomes). The Jaccard similarity coefficient is calculated as s/(u1 + u2 + s), where s is the number of similar outcome definitions the 2 trials share, and u1 and u2 are the number of outcome definitions that are unique to each of the trials. The average Jaccard similarity coefficient is the arithmetic mean of coefficients for all possible combinations of 2 trials.

| **Concept of interest**  **(N trials)** | **Number of outcomes reported in the included trials assessing the concept of interest (n=464)** | **Number of different outcome definitions**  **(n=261)** | **Average Jaccard similarity coefficient** |
| --- | --- | --- | --- |
| Diabetes control (n=21) | 266 | 153 | 10% |
| Assessment of diabetic foot complications (n=1^a^) | 2 | 2 | -^b^ |
| Physical activity (n=23) | 87 | 46 | 18% |
| Blood pressure control (n=5) | 32 | 13 | 32% |
| Adherence to treatment (n=5) | 22 | 13 | 7% |
| Heart rate variability (n=4) | 14 | 14 | 0% |
| Sleep disturbance(n=2^a^) | 39 | 20 | 48% |
| Pulmonary capacity (n=1) | 2 | 2 | -^b^ |

^a^ Trials could assess multiple concepts of interest (e.g., diabetes control and diabetic foot complications; physical activity and sleep disturbance). ^b^ Only one trial assessed diabetic foot complications and pulmonary capacity.

**Appendix 4: Outcome definitions measured with biometric monitoring devices (BMDs) for adherence to treatment, blood pressure control, pulmonary capacity, heart rate variability, diabetic foot assessment and sleep disturbance.** Each node represents an outcome definition. The size of nodes represents the number of times each outcome definition was used in the included trials. Outcome definitions are clustered by outcome domains.


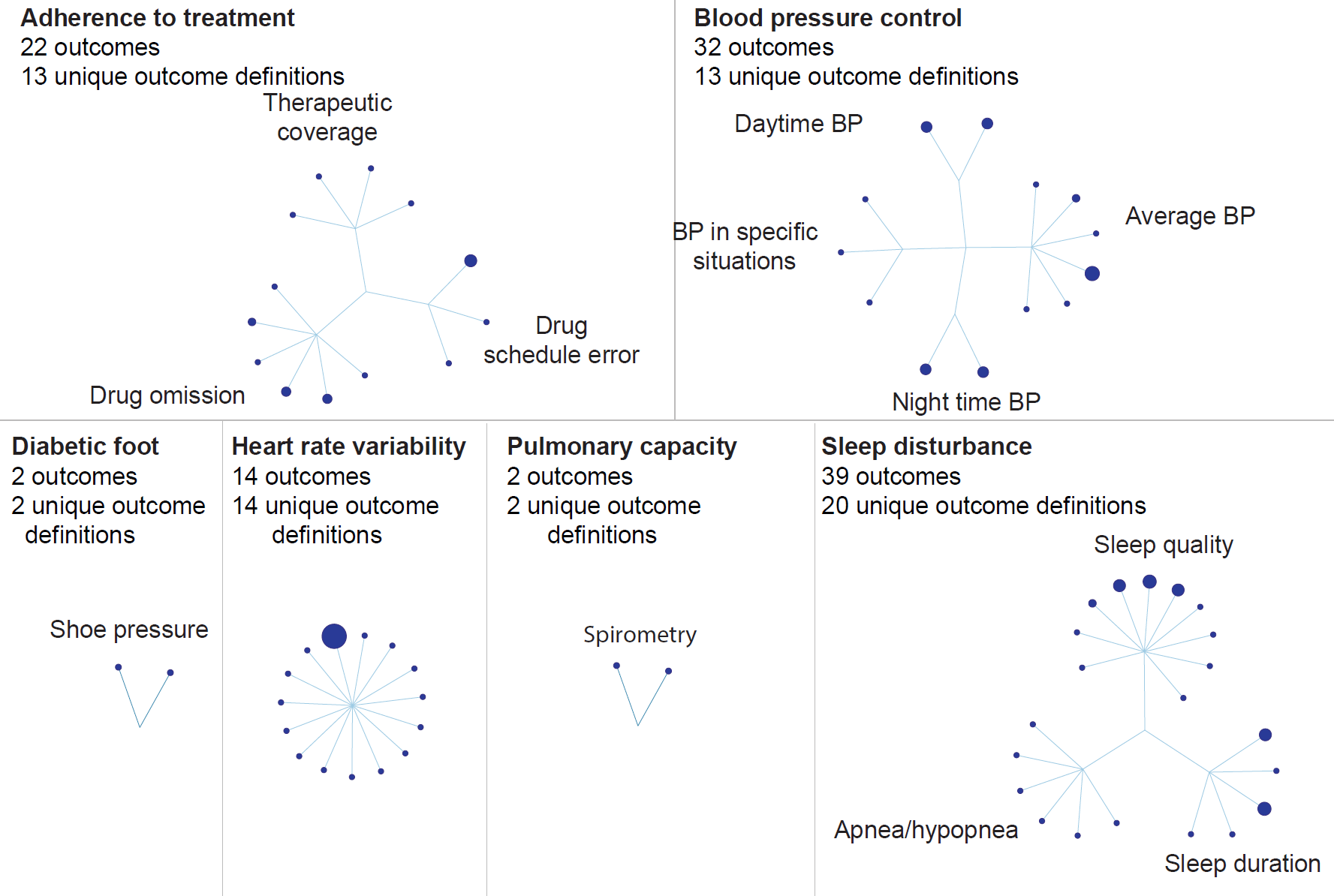


**Appendix 5: List of unique definitions of outcomes measured with BMDs.**

| **Diabetes control**   1. 00.00-06.00 hours AUC < 3.9 mmol/L 2. 00.00-06.00 hours mean glucose level 3. 00.00-06.00 hours M-value (target glucose level 90 mg/dl) 4. 00.00–06.00 hours M-value (target glucose level = 90) 5. 00.06-12.00 hours M-value (target glucose level 120 mg/dl) 6. 06.00-24.00 hours mean glucose level 7. 08.00-12.00 hours AUC < 3.9 mmol/L 8. 08.00-12.00 hours mean glucose level 9. 08.00-12.00 hours standard deviation 10. 08.00–12.00 hours M-value (target glucose level = 120) 11. 1-h before breakfast mean glucose concentration 12. 1-h before dinner mean glucose concentration 13. 1-h before lunch mean glucose concentration 14. 1-h postmeal relative area under the continuous glucose monitoring curve at breakfast 15. 1-h postmeal relative area under the continuous glucose monitoring curve at dinner 16. 1-h postmeal relative area under the continuous glucose monitoring curve at lunch 17. 12.00-24.00 hours mean glucose level 18. 12.00-24.00 hours M-value (target glucose level 120 mg/dl) 19. 12.00-24.00 hours standard deviation 20. 12.00–24.00 hours M-value (target glucose level = 120) 21. 2-h postmeal relative area under the continuous glucose monitoring curve at breakfast 22. 2-h postmeal relative area under the continuous glucose monitoring curve at dinner 23. 2-h postmeal relative area under the continuous glucose monitoring curve at lunch. 24. 24-h mean glucose level 25. 24-h mean weighted glucose 26. 24-h M-value (target glucose level 100 mg/dL) 27. 24-h to 8 h mean glucose level 28. 3-h postmeal relative area under the continuous glucose monitoring curve at breakfast 29. 3-h postmeal relative area under the continuous glucose monitoring curve at dinner 30. 3-h postmeal relative area under the continuous glucose monitoring curve at lunch. 31. 4-h postmeal relative area under the continuous glucose monitoring curve at breakfast 32. 4-h postmeal relative area under the continuous glucose monitoring curve at dinner 33. 4-h postmeal relative area under the continuous glucose monitoring curve at lunch 34. 8 h to 24-h mean glucose level 35. AUC > 10 mmol/L 3 h after breakfast 36. AUC > 10 mmol/L 3 h after dinner 37. AUC > 10 mmol/L 3 h after lunch 38. AUC of glucose <2.5 mmol/l during 24 h 39. AUC of glucose <3.1mmol/l during 24 h 40. AUC of glucose <3.3 mmol/L during 24 h 41. AUC of glucose <3.5 mmol/L during 24 h 42. AUC of glucose <3.9 mmol/l during 24 h 43. AUC of glucose >0 mmol/L during 24 h 44. AUC of glucose >10 mmol/L during 24 h 45. AUC of glucose >3.9 and <10 mmol/l during 24 h 46. AUC> 8.9 mmol/L 3 h after breakfast 47. AUC> 8.9 mmol/L 3 h after dinner 48. AUC> 8.9 mmol/L 3 h after lunch 49. Between-day coefficient of variation in glucose level 50. Blood glucose risk index 51. Coefficient of glucose variation during day time (06.00-24.00 hours) 52. Coefficient of glucose variation during night-time (00.00-06.00 hours) 53. Coefficient of variation of 24-h glucose 54. Continuous overall net glycemic action (CONGA) 2 h 55. Continuous overall net glycemic action (CONGA) 6 h 56. Daily risk range 57. Distance traveled in 24 h 58. Duration of glucose level above target (> 10 mmol/L) 59. Duration of glucose level below target (< 3.0 mmol/l ) 60. Duration of glucose level below target (<3.9 mmol/L) 61. Duration of glucose level was in a target range (3.9–10 mmol/L) 62. Excursion frequency 24 h 63. Fasting glucose at 6.00 64. Frequency of nocturnal hypoglycemia 65. Highest postprandial glucose level within 3 h after breakfast 66. Highest postprandial glucose level within 3 h after lunch 67. Highest postprandial glucose level within 3 h after supper 68. Incremental area under the concentration-time curve 2 h after breakfast 69. Incremental area under the concentration-time curve 2 h after dinner 70. Incremental area under the concentration-time curve 3 h after lunch 71. incremental area under the curve for 1900–0700 h 72. Incremental area under the curve > 4 h after dinner 73. Incremental area under the curve > 4 h after lunch 74. Large amplitude of glycemic excursion (LAGE) 75. Low blood glucose index (LBGI) 76. Maximum blood glucose 77. Mean amplitude of glycemic excursion (MAGE) of decreased glucose levels 78. Mean Amplitude of Glycemic excursions (MAGE) of increased glucose levels 79. Mean Amplitude of Glycemic excursions (MAGE) 80. Mean of daily difference (MODD) 81. Mean plasma/interstitial fluid glucose gradient 82. Minimum blood glucose 83. Number of events of glucose <2.2mmol/l in 24 h 84. Number of events of glucose <2.5 mmol/l during night time (23.00-06.00 hours) 85. Number of events of glucose <2.5 mmol/l in 24 h 86. Number of events of glucose <3.0 mmol/l during night time (00.00-06.00 hours). 87. Number of events of glucose <3.0 mmol/l in 24 h 88. Number of events of glucose <3.1 mmol/l during night time (23.00-06.00 hours). 89. Number of events of glucose <3.1 mmol/l in 24 h 90. Number of events of glucose <3.9 mmol/L during night period (23.00-06.00 hours) 91. Number of events of glucose <3.9 mmol/l in 24 h 92. Number of hours of glucose <2.5 mmol/l during 24 h 93. Number of hours of glucose <2.5 mmol/l during night time (23.00-06.00 hours) 94. Number of hours of glucose <3.1 mmol/l during 24 h 95. Number of hours of glucose <3.1 mmol/l during night time (23.00-06.00 hours) 96. Number of hours of glucose <3.9 mmol/l during night time (23.00-06.00 hours) 97. Number of hours of time in glucose <2.2 mmol/l during 24 h 98. Number of sensor scans/day 99. Peak height at breakfast (mmol/l) (postprandial glucose peak–premeal glucose) 100. Peak height at dinner (mmol/l) (postprandial glucose peak–premeal glucose) 101. Peak height at lunch (mmol/l) (postprandial glucose peak–premeal glucose) 102. Percentage of glucose values below target (< 3.0 mmol/L) 103. Percentage of glucose values below target (< 3.9 mmol/L) 104. Percentage of time glucose level was below target (< 5mmol/L) 105. Percentage of time glucose level was in a target range (3.9–10 mmol/L) 106. Percentage of time glucose level was in a target range (3.9–10 mmol/L) 24 h to 8 h 107. Percentage of time glucose level was in a target range (3.9–10 mmol/L) 8 h to 24 h 108. Percentage of time glucose level was in a target range (3.9–10 mmol/L) after 2 h from meal start 109. Percentage of time glucose level was in a target range (5.5–10 mmol/L) 110. Percentage of time glucose level was in a target range (glucose 5–10 mmol/L) 111. Percentage of time of glucose level above target (> 10 mmol/L) 112. Percentage of time of glucose level above target (> 15 mmol/L) 113. Percentage of time of glucose level above target (> 20 mmol/L) 114. Percentage of time of glucose level above target (>13.3 mmol/L) 115. Percentage of time of glucose level above target (>13.9 mmol/L) 116. Percentage of time of glucose level above target (>16.7 mmol/L) 117. Percentage of time of glucose level below target (< 5.5 mmol/L) 118. Percentage of time of glucose level below target (<2.8 mmol/L) 119. Percentage of time of glucose level below target (<3.0 mmol/L) 120. Percentage of time of glucose level below target (<3.5 mmol/L) 121. Percentage of time of glucose level below target (<3.5 mmol/L) 24 h to 8 h 122. Percentage of time of glucose level below target (<3.5 mmol/L) 8 h to 24 h 123. Percentage of time of glucose level below target (<3.9 mmol/L) 124. postprandial glucose gradient at breakfast 125. postprandial glucose gradient at lunch 126. postprandial glucose gradient at supper 127. Postprandial glucose levels after breakfast 128. Postprandial glucose levels after dinner 129. Postprandial glucose levels after lunch 130. Postprandial glucose peak at breakfast 131. Postprandial glucose peak at dinner 132. Postprandial glucose peak at lunch 133. Preprandial glucose level lunch-breakfast 134. Preprandial glucose levels before breakfast 135. Preprandial glucose levels before dinner 136. Preprandial glucose levels before lunch 137. Range of glucose increase from pre-meal to postprandial peak levels at breakfast 138. Range of glucose increase from pre-meal to postprandial peak levels at dinner 139. Range of glucose increase from pre-meal to postprandial peak levels at lunch 140. Standard deviation (SD) of 24-h glucose levels 141. SD of 24-h to 8-h glucose levels 142. Standard Deviation (SD) of 8-h to 24-h glucose levels 143. Standard Deviation (SD) of daytime (00.00-6.00 hours) glucose levels 144. Standard Deviation (SD) of daytime (06.00-24.00 hours) glucose levels 145. System utilization 146. Time from start of meal to the highest postprandial glucose level (min) breakfast 147. Time from start of meal to the highest postprandial glucose level (min) dinner 148. Time from start of meal to the highest postprandial glucose level (min) lunch 149. Time of glucose values > 13.9 mmol/L in the previous 4 h 150. Time to postprandial peak glucose levels after breakfast 151. Time to postprandial peak glucose levels after lunch 152. Time to postprandial peak glucose levels from before after dinner 153. Total energy (product of the sum of squared frequency and the amplitude of Fourier coefficients for the 24-h individual average tissue glucose curves)   **Assessment of diabetic foot complications**   1. In-shoe peak pressure at ulcer 2. Different of peak pressure at ulcer site in device compared to patient's shoe   **Physical activity**   1. Average active time /day 2. Average active time /week 3. Average daily sitting time 4. Average daily standing time 5. Average daily stepping time 6. Average number of steps/activities/day 7. Average number of steps/activities/days 8. Average number of steps/activities/week 9. Average walking intensity 10. Distance/ week 11. Distance/day 12. Duration of lying time/day 13. Duration of sedentary behavior/day 14. Duration of sedentary behavior/week 15. Duration of sedentary bouts/day 16. Duration of vigorous physical activity/day 17. Floors per day 18. Floors per week 19. Metabolic equivalent of task (MET) 20. Number of bouts/week 21. Number of 30 min sitting bouts/day 22. Number of 60 min sitting bouts/day 23. Number of days ≥ 30 min of physical activity 24. Number of days wearing the device 25. Number of patients that walked at least 6500 steps/day 26. Number of sedentary bouts/week 27. Number of sit-to-stand transitions/day 28. Percentage of wear time in moderate-to-vigorous physical activity 29. Percentage of wear time in prolonged moderate-to-vigorous physical activity 30. Percentage of wear time in sedentary behavior 31. Percentage of wear time lying 32. Percentage of wear time sitting 33. Physical activity composite (weekly minutes of moderate-to-vigorous physical activity (MVPA) and average steps/day) 34. Physical activity energy expenditure 35. Sitting time per day ≥ 30 min 36. Sitting time per day ≥ 60 min 37. Time in low activity/day 38. Time in low activity/week 39. Time in low-moderate activity/day 40. Time in low-moderate activity/week 41. Time in moderate activity/week 42. Time in moderate to vigorous physical activity/day 43. Time in vigorous activity/day 44. Total daily energy expenditure 45. Total time in bouts/week 46. Walking time per day   **Blood pressure (BP) control**   1. 24-h mean systolic BP 2. 24-h mean diastolic BP 3. Proportion of patients achieving average 24-h diastolic BP <80 mmHg 4. Proportion of patients achieving average 24-h systolic BP <130 mmHg 5. Proportion of patients achieving average daytime BP <135/85 mmHg and nighttime BP <120/70 mmHg 6. Daytime mean systolic BP 7. Daytime mean diastolic BP 8. Night-time mean diastolic BP 9. Night-time mean systolic BP 10. Mean sitting diastolic BP 11. Mean sitting systolic BP 12. Proportion of patients achieving average 24-h BP <130/80 mmHg 13. Percentage of non-dippers (patients with ±10% change in night-time BP from daytime BP)   **Sleep quality**   1. Apnea/Hypopnea Index (AHI) 2. Fragmentation index 3. Mean O2 saturation 4. Minimal O2 saturation 5. Movement index 6. Oxygen Desaturation Index (ODI) 7. Respiratory Disturbance Index (RDI) 8. Rise time 9. Sleep efficiency (whole time in bed) 10. Sleep efficiency in the first third of time in bed 11. Sleep efficiency in the second third of time in bed 12. Sleep efficiency in the third of time in bed 13. Sleep offset time 14. Sleep onset latency 15. Time in bed 16. Time spent in O2 saturation 17. Time spent in stage N3 sleep measured in minutes 18. Time spent in stage REM sleep measured in minutes 19. Total sleep time 20. Wake after sleep onset   **Adherence to treatment**   1. Correct interdose interval 2. Missed dose (% of prescribed doses taken >28 h after the previous one or > 14 h after the previous one) 3. Number of unprotected days 4. Number of days with the wearable worn 5. Overall adherence (number of container openings/number of prescribed doses during the period) 6. Percentage correct time>80% 7. Percentage of adherent patients (>80% doses taken) 8. Percentage of correct days >80% 9. Percentage of days with correct number of doses taken 10. Percentage of doses taken 11. Percentage therapeutic cover 12. Percentage therapeutic cover>80% 13. Therapeutic coverage (mean percentage of the study period covered with drug activity)   **Pulmonary capacity**   1. Forced expiratory volume 2. Forced vital capacity   **Heart rate variability**   1. Absence of arrhythmia during 6/7th time 2. Heart rate variability–low frequency 3. Heart rate variability–high frequency 4. Heart rate variability–low frequency/high frequency ratio 5. Incidence of newly diagnosed AF at the end of the 12-month monitoring period 6. Incidence of newly diagnosed AF at the end of the 4-month monitoring period 7. Mean heart rate 8. Mean value of RR intervals 9. Number of episodes with arrhythmia 10. Standard deviation of normal intervals 11. Standard deviation of the mean of RR intervals in successive 5-min segments 12. Mean of the standard deviation of all normal RR-intervals for all 5-min segments 13. Square root of the mean of the sum of the squares of differences between adjacent intervals 14. Triangular index |
| --- |

AUC, area under the receiver operating characteristic curve; AF, atrial fibrillation; BP, blood pressure
